# Implementation mapping for tobacco cessation in a federally qualified health center

**DOI:** 10.3389/fpubh.2022.908646

**Published:** 2022-09-02

**Authors:** Ariel M. Domlyn, Carolyn Crowder, Howard Eisenson, Kathryn I. Pollak, James M. Davis, Patrick S. Calhoun, Sarah M. Wilson

**Affiliations:** ^1^VA Health Services Research and Development Center of Innovation to Accelerate Discovery and Practice Transformation, Durham, NC, United States; ^2^Lincoln Community Health Center, Durham, NC, United States; ^3^Department of Family Medicine and Community Health, Duke University School of Medicine, Durham, NC, United States; ^4^Department of Population Health Sciences, Duke University School of Medicine, Durham, NC, United States; ^5^Duke Cancer Institute, Duke University Health System, Durham, NC, United States; ^6^Department of Medicine, Duke University School of Medicine, Durham, NC, United States; ^7^Department of Psychiatry and Behavioral Sciences, Duke University School of Medicine, Durham, NC, United States

**Keywords:** implementation science (MeSH), implementation mapping, tobacco cessation, 5A's smoking cessation guidelines, community health, community-engaged dissemination and implementation

## Abstract

**Background:**

Implementation mapping (IM) is a promising five-step method for guiding planning, execution, and maintenance of an innovation. Case examples are valuable for implementation practitioners to understand considerations for applying IM. This pilot study aimed to determine the feasibility of using IM within a federally qualified health center (FQHC) with limited funds and a 1-year timeline.

**Methods:**

An urban FQHC partnered with an academic team to employ IM for implementing a computerized strategy of tobacco cessation: the 5A's (Ask, Advise, Assess, Assist, Arrange). Each step of IM was supplemented with theory-driven methods and frameworks. Data collection included surveys and interviews with clinic staff, analyzed *via* rapid data analysis.

**Results:**

Medical assistants and clinicians were identified as primary implementers of the 5A's intervention. Salient determinants of change included the perceived compatibility and relative priority of 5A's. Performance objectives and change objectives were derived to address these determinants, along with a suite of implementation strategies. Despite indicators of adoptability and acceptability of the 5A's, reductions in willingness to adopt the implementation package occurred over time and the intervention was not adopted by the FQHC within the study timeframe. This is likely due to the strain of the COVID-19 pandemic altering health clinic priorities.

**Conclusions:**

Administratively, the five IM steps are feasible to conduct with FQHC staff within 1 year. However, this study did not obtain its intended outcomes. Lessons learned include the importance of re-assessing barriers over time and ensuring a longer timeframe to observe implementation outcomes.

## Introduction

Community-engaged dissemination and implementation (CEDI) research is a process of collaboration and shared decision-making between academics and community-based healthcare providers and recipients (1). CEDI is presumed to mitigate health inequities by incorporating the perspectives of individuals typically marginalized from traditional research paradigms (1, 2). Implementation Mapping (IM) (3) is a CEDI method with growing popularity (2, 3). IM hybridizes implementation science principles with a process for multi-level health promotion called intervention mapping. IM defines five change management steps (3). Despite being touted as a promising strategy (4, 5) and multiple examples of planned use *via* study protocols (4, 6, 7) there are few publicly accessible descriptions of applying the IM process (8–10) and among these only one reported use through all steps (8). This complete example effectively illustrates IM as a feasible and effective method, however, it was also bolstered by significant resources (a 4-year timeline and five funding sources). We offer an example of using IM on a smaller scale within a busy, urban federally qualified health center (FQHC). The lessons learned from this pilot study offer perspective on the feasibility (11) of conducting IM in resource-limited settings.

## Materials and methods

Tobacco cessation is an important public health effort (12). Despite declining rates of tobacco use in recent years, tobacco rates among low-income individuals remain unchanged (13). Community clinics and primary care providers are front line forces for the prevention and treatment of harmful health behaviors, including tobacco use. This project sought to use IM to implement an evidence-based tobacco cessation strategy within a community healthcare center. Table 1 provides the definitions of terms used throughout this text.

**Table 1:** Definitions of key terms.

**Term**	**Definition**
5A	Ask, Advise, Assess, Assist, Arrange: A health provider-delivered tobacco cessation strategy.
Adopters	Decision makers who hold power to decide whether an innovation is adopted; in this example, clinic leaders like medical chiefs.
CEDI	Community-Engaged Dissemination and Implementation: a process of collaboration and shared decision-making between academics and community-based healthcare providers and recipients.
CFIR	Consolidated Framework for Implementation Research: a comprehensive model composed of determinants with empirical and theoretical support for implementation relevance, such as characteristics of the intervention, inner setting, and outer setting.
Change objectives	The behaviors necessary for each FQHC staff role to exhibit in order to successfully implement an innovation.
Determinants	Barriers and facilitators of successfully implementing the innovation.
EHR	Electronic Health Record: a digital system for managing patient health information.
EPIS	Exploration, Planning, Implementation, Sustainment: A four-stage conceptualization of the implementation process.
FQHC	Federally Qualified Health Center: Community-based health clinic that receives US federal funds for providing primary care services to underserved areas.
IM	Implementation mapping: A five-step change management process.
Implementation outcomes	Expected and observed indicators of successful innovation adoption, usage, and maintenance. These are markers of interim progress and may be assessed early, mid, or late in the project.
Implementers	Individuals responsible for regularly executing the innovation to ensure it becomes routine practice; in this example, healthcare providers.
Innovation	A policy, program, or process new to the setting, alternatively referred to as an intervention; in this example, the 5As.
Performance objectives	Tasks that define the specific steps or behaviors needed to obtain implementation outcomes.

### Setting: Federally qualified health center

Nationally, tobacco use rates are highest among those at or below 200% of the federal poverty level (13). In Durham, North Carolina tobacco use remains a leading cause of death in the area (14). At one local FQHC, 97% of patients have income at or below 200% of the federal poverty level (15). This FQHC serves over 34,000 adult and pediatric patients per year. In 2015, the FQHC attempted to implement an evidence-based specialty tobacco cessation clinic with trained tobacco treatment specialists. Despite early successes, the program was not sustained due to staff turnover. To address this concern, the FQHC's director of behavioral health (CC) partnered with an academic with expertise in implementation science and clinical psychology (SW) to address patient tobacco use and design a sustainable program.

The present project sought to create a package of implementation strategies designed to facilitate uptake and sustainment of an evidence-based, technology-assisted tobacco cessation tool at the FQHC. In consultation with clinician and researcher colleagues, the CEDI leadership team selected computer-facilitated delivery of evidence-based 5A's due to its known impact increasing delivery of tobacco cessation treatment in medical settings (16, 17).

### Intervention: 5A's intervention model for tobacco cessation

The 5A's intervention model (Ask, Advise, Assess, Assist, Arrange) was developed as a guide to help clinicians treat tobacco use (12). One method proposed to facilitate clinician use of the 5A's is to use a computerized process with handheld digital devices (16–18). While this strategy has proven effective, there are some implementation issues with introducing handheld devices into clinical encounters where they are not normally used (16). The present study sought to overcome implementation barriers to computerized 5A's by implementing this evidence-based intervention into the electronic health record (EHR) system at an FQHC. This would enable the 5A's to be completed with fidelity directly through the EHR rather than using any outside devices or manuals. However, it is recognized that technology-assisted smoking cessation tools may suffer significant challenges in implementation including limited staff knowledge of resources, limited familiarity with tobacco cessation practices, and lack of organizational support (16, 19). Numerous factors (20) may affect uptake and sustainment, including disruption of clinic workflow, as well as perceptions that technology is burdensome and ineffective (21, 22). Systematic implementation planning and support may improve uptake and sustainment of technology-dependent tobacco cessation interventions. In selecting an implementation method, the implementers prioritized equity-focused options that accounted for situations unique to community-academic collaborations.

### CEDI method

Use of CEDI methods are critical in FQHC settings, as patients served by these clinics are often among the most disenfranchised (23). Derived from literatures on health promotion and implementation science, implementation mapping (IM) is a CEDI process that includes five steps for assisting organizations in planning and enacting change strategies. The steps detail (1) conducting a needs and assets assessment within the setting, (2) identifying implementation outcomes and performance objectives based on identified change determinants, (3) selecting a theory-based method and strategies to affect these determinants, (4) developing implementation protocols and materials, (5) evaluating implementation outcomes (3). Standardized measures or tools are not yet available for enacting each step, but guidelines exist to inform the process. Key among these is the use of theory to inform each step (3).

The current project used the Consolidated Framework for Implementation Research (CFIR) (24) to design needs assessment materials and identify determinants of change. Determinants are the barriers and facilitators affecting whether the innovation is adopted, scaled, and maintained; these are classified into discrete constructs related to the implementation process or the innovation itself (24). CFIR is a comprehensive model composed of determinants with empirical and theoretical support for implementation relevance, such as characteristics of the intervention, inner setting, and outer setting. This includes knowledge (staff familiarity with the innovation), compatibility (perceived fit between the innovation and organization), relative priority (perceived importance of the innovation), and the implementation climate (staff receptivity to the innovation) (24).

For designing implementation strategies (methods or techniques used to enhance the adoption, implementation, and sustainability of a clinical program or practice) (25, 26), a systems science method (27) was used to assess the variable impact and effort of each potential strategy and adapted for developing implementation strategies within this FQHC (28). Consistent with community-engaged practices (2), this process enabled power-sharing by identifying staff-driven strategies, later mapped onto a taxonomy of expert-identified strategies (26) for consistency in reporting.

A well established conceptualization of implementation outcomes (29) assisted in reporting progress for the final step. Unlike service or population outcomes, implementation outcomes include both expected and observed indicators of successful innovation adoption, usage, and maintenance (30). These types of outcomes identify markers of interim progress in the implementation efforts and may be assessed temporally early, mid, or late in the project (29). Debate about conceptualizing implementation outcomes (30) unfolded in the literature within the timeframe this pilot study was conceptualized and executed. Reporting for step 5 considers the anticipated implementation outcomes as perceived acceptability [degree of satisfaction or palatability of the innovation (29)] and adoptability [the likelihood key decision-makers will decide to put the innovation into place (30)]. Actual implementation outcomes are adoption [the extent key decision makers decide to put the innovation into place (30)] and implementation [the extent the innovation is in place (30)]. Of note: throughout this paper the word “feasibility” refers to the common term for a preparatory study (11) rather than the “feasibility” as defined in implementation outcomes (29).

### Data collection and analysis

Participants included multiple groups of FQHC clinical and administrative staff: physicians, advanced practice providers, behavioral health specialists, nurses, medical assistants, patient educators, and administrative and clinical leaders. Inclusion of different clinic roles aimed for diversity of opinions to generate staff-driven solutions. We used quota sampling (31) to ensure representation across clinic roles (physicians, advanced practice providers, behavioral health specialists, nurses, medical assistants, and patient educators) and settings (internal medicine, family medicine, and pediatrics). All clinic staff members who contacted the study coordinator for participation were included in the study. Participants (*N* = 12) were interviewed using open-ended prompts for the needs assessment and determinant identification. This sample size was selected given its high likelihood of reaching data saturation (51). These interviews were recorded, transcribed, and analyzed using a rapid analytic method in which data reduction occurred prior to coding (32). Concept codes were determined *a priori* with the goal to rapidly inform process (33). Interview results informed Step 4, the development of implementation protocols and materials. Surveys were then conducted with FQHC clinic and administrative leaders (*N* = 7), and descriptive statistics reported to identify performance objectives and gather early-stage implementation outcomes. Informed consent was obtained for all participants. Only non-FQHC study staff had access to identifying information of staff participants. All FQHC investigators saw only de-identified, aggregated data.

**Figure 1:**
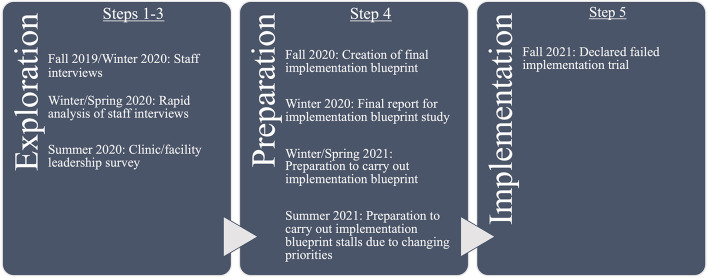
Project timeline.

Figure 1 shows the project timeline by key activity, IM step, and implementation stage. Key activities here are data collection, analysis, and development of materials. Implementation stages are discerned from a common stage framework that determines the stages of change that occur within an organization: Exploration, Preparation/Adoption, Implementation, and Sustainment (EPIS) (47). Both IM and EPIS are heuristics for describing the process, but both can be iterative rather than linear processes. Therefore, key activities do not always occur sequentially according to these steps and stages.

## Results

Over the course of 12 months, all five IM steps were planned and executed iteratively, as described in the original IM process (3). However, not all objectives and outcomes were achieved. Results below illustrate the process as it was planned and unfolded, but readers are advised to note that the implementation package described in Step 4 was not adopted, and therefore the planned activities and objectives in Step 3 were not undertaken. In Step 5, anticipated outcomes showed promising indicators of eventual actual implementation outcomes; this is further described in that step.

### IM step 1: Needs assessment

Implementation adopters were identified by soliciting opinions about the most appropriate staff member to oversee the process of rolling out the 5A's tool in the FQHC's EHR. In IM, implementation adopters are the decision makers, such as leaders, who hold power to decide whether an innovation is adopted (3). There was not clear consensus on this question, with most staff endorsing multiple possible adopters, generally among those who already held clinic leadership positions. Most staff endorsed either the clinical chief for each department or the head of behavioral health. Three interviewees (25%) suggested that providers be the ones to decide to adopt the intervention.

Once implementation adopters are identified, IM indicates adopters be involved in the subsequent planning process. Throughout planning, preparation, and implementation there was shared decision-making and collaboration between adopters (CC, HE) and academic partners (SW, JD, PC), in activities including brainstorming sessions, planning meetings, and addressing issues around participant selection, qualitative methodology, selection of implementation framework, and identifying staff engagement strategies.

Choices of appropriate implementers varied across the 5A's steps. Implementers, per IM, are the individuals responsible for regularly executing the innovation and ensuring it becomes routine practice (3). Clinic staff interviewees (*N* = 12) were given options to endorse one or more roles for each step, thus number of endorsements exceeded the number of interviewees. Clinic staff unanimously identified medical assistants as having the knowledge and skills to conduct the first 5A's step of asking patients about tobacco use. Per the second 5A's step of advising on tobacco cessation, most (*N* = 11) staff stated that medical providers were the most appropriate implementers. Concerning the third 5A's step (assessing patient willingness to quit) several staff identified multiple potential implementers. Most staff endorsed that medical providers should conduct the motivational interviewing (34) necessary for this step, yet there were also four mentions each of behavioral health providers and medical assistants being capable of conducting this portion of the intervention. The fourth 5A's step (assist the patients to quit) was seen as a joint effort between medical and behavioral health providers, with nine endorsements of medical providers conducting this step and prescribing nicotine replacement therapies, and six endorsements of behavioral health support as necessary for counseling or consultation. No staff suggested medical assistants as implementers for this step. For the fifth 5A's step (following up with patients), staff were divided on the optimal implementers. There was equal endorsement for the medical provider, behavioral health provider, and whomever was conducting the primary intervention (e.g., pharmacotherapy, counseling, etc.). Responses at this step were contingent upon who the interviewee had identified as the primary responsible party in the previous step. Collectively, identified implementers of the 5A's intervention were clinical staff members, particularly medical assistants, nurses, primary care providers, and behavioral health providers.

### IM step 2: Identify adoption and implementation outcomes, performance objectives, determinants, and change objectives

#### Performance objectives and implementation outcomes

Performance objectives were derived from surveys administered to FQHC leadership (*N* = 7) and from follow-up meetings with the CEDI leadership team (CC, SW, HE, PC, JD). These objectives intend to define the specific steps or behaviors needed to obtain implementation outcomes. Performance objectives gleaned from leadership surveys are displayed in Table 2.

**Table 2:** Performance objectives and implementation outcomes.

**Target role**	**Implementation outcomes**	**Performance objectives (tasks/behaviors)**
Medical chiefs: Adopter	Decide to adopt the 5A's intervention package for integration into EHR.	1. Agree to integrate 5A's into clinical care 2. Agree to integrate 5A's into EHR 3. Dedicate time for clinic staff training 4. Gain support from clinic staff
Medical assistants: Implementer (5A steps 1–2)	Complete first two steps of 5A's intervention, appropriately document and communicate to clinicians.	1. (Ask) Ask whether patient is a current or past tobacco user, then classify in EHR 2. Communicate results to clinician.
Clinician (physicians, advanced practice providers): Implementer (5A steps 3–5)	Receive information completed by Medical Assistants, complete final three steps of 5A's intervention, appropriately document.	1. (Assess) Assess if the patient is willing to make an attempt to quit tobacco use. Document in EHR. 2. (Advise) If current user, advise patient to quit using clear and personalized manner. Document in EHR. 3. (Assist) Use brief motivational interviewing to increase likelihood of quit attempt. Deliver appropriate prescription. Document in EHR. 4. (Arrange) Refer to behavioral health or state quitline as needed. Schedule follow-up visit as needed. Select tobacco use after-visit summary with information on free cessation resources. Document in EHR.
Behavioral health chief: Maintainer	Leverage relationship with clinic leadership to ensure ongoing evaluation and quality improvement of 5A's process.	1. Talk with clinic leadership about implementation plans and concerns. 2. Participate in the planning team. 3. Advocate for ongoing time and resources for assisting implementers.

#### Determinants

Interviewers asked FQHC staff open-ended questions pertaining to potential determinants [i.e., barriers and facilitators of successfully implementing the intervention (24)]. These determinants included staff knowledge of the 5A's intervention, compatibility of 5A's with current clinic practices, implementation climate (i.e., staff receptivity to 5A's), and relative priority of implementing 5A's. Most (*N* = 9) staff reported no familiarity with the 5A's intervention, while the remaining staff (*N* = 3) stated they had a vague recollection of having learned this previously, such as in graduate training. All reported percieving the 5A's as compatible with current practices, and several staff said they routinely one or more of the steps as part of usual care. One respondent clarified that they would be opposed to the process if it were mandated, preferring it to be optional and limited to patients who were known tobacco users. Two respondents indicated more information would improve perceived compatibility, such as further education on the 5A's or seeing evidence of the innovation's efficacy in other clinics. Five respondents noted that one barrier to compatibility is the perceived burden of time and effort to conducting the 5A's, which could be mitigated by streamlining the documentation process. Regarding implementation climate, the majority (*N* = 8) of respondents were in favor of integrating the 5A's into the EHR, some expressing strong optimism about its potential. Three interviewees expressed neutral or ambivalent sentiment, such as: “Adding [this to the] chart is both great and challenging. [There are already] so many other things to [the EHR].” One staff member at the pediatric clinic was opposed to the innovation, stating that they already had a template for asking teenagers about smoking and thought the yield would be low in this population. Per the perceived priority, three participants indicated the tobacco cessation intervention was a priority while four staff members indicated it was a low priority. Six respondents did not state whether it seemed like a priority: five of those indicated the intervention seemed feasible and one stated it would depend on the clinic flow.

#### Change objectives

Change objectives were developed by cross-walking previously identified performance objectives with determinants of change. Change objectives are the behaviors necessary for FQHC staff to exhibit in order to successfully implement the 5A's. In Table 3, a sample of change objectives is shown with columns corresponding to the necessary change in attitudes, knowledge, and skills for various FQHC roles. Each cell lists an observable behavior that would be indicative of a change in attitude, knowledge, or skills. These are marked by the expectation that each change objective would affect the perceived compatibility of 5A's or the perceived relative priority of 5A's.

**Table 3:** Change objectives by implementation role.

**Role**	**Change objectives**		
	**Attitude**	**Knowledge**	**Skills**
Medical chiefs: Adopter	P: Express importance of addressing tobacco use P: Express that 5A's process is everyone's job C: Express ease of use with computerized process	P: Clarify each staff member's role in the process C: Clarify process and room for flexibility within existing clinic workflows	C: Monitor success in implementation using data for audit and feedback
Medical Assistants: Implementer (5A steps 1-2)	P: Express importance of addressing tobacco use C: Identify parts of 5A's that are already routine practice C: Express ease of use with computerized process	P: Explain role of tobacco use for long-term health outcomes P: Describe number of patients who are tobacco users P: Note differences in 5A's depending on age of patient C: Explain role in 5A's process	C: Demonstrate ability to use computerized 5A's process, including locating and entering patient tobacco use information into EHR fields C: Demonstrate ability to notify appropriate provider(s) of next steps in 5A's
Clinician (physicians and advance practice providers): Implementer (5A steps 3–5)	P: Express importance of addressing tobacco use P: Express pro/cons of 5A's process C: Identify parts of 5A's that are already routine practice C: Express ease of use with computerized process	P: Note differences in 5A's depending on age of patient P: Explain why early intervention is important for health outcomes C: Explain interventions for tobacco use by type (e.g., combustible, vaping, dip/chew) C: Explain amount of time expected for 5A's process C: Explain role in 5A's process (e.g., prescribing, referring) C: Explain information to be included in patient after-visit summary	C: Demonstrate ability to use computerized 5A's process, including locating and entering patient tobacco use information into EHR fields C: Demonstrate use of age-appropriate brief behavioral interventions (e.g., MI) for tobacco use C: Demonstrate ability to successfully prescribe tobacco cessation medications C: Demonstrate ability to provide referral options

C, Compatibility; P, Relative Priority.

### Step 3: Select theoretical methods and design implementation strategies

The selection of implementation strategies requires identifying techniques to influence determinants gleaned from the previous step. There is much debate in the literature about best methods for selecting strategies, with general consensus that a systematic and constituent-influenced approach is optimal, with the entire IM process often cited as an option (35, 36). Here, a three-component approach was adapted from the effort-vs-impact assessment method of operations planning, fully described elsewhere (28). In brief, this approach charted strategies according to effort (low/high) and impact (low/high). The first component assessed the potential effort to make the technological strategy usable according to availability (i.e., how accessible the technological infrastructure is to clinic staff) and familiarity (i.e., how much training would be required for staff). The second component assessed potential impact of the strategy (i.e., improving monitoring, communication, or data collection). The third component assessed whether to use or abandon the strategy by cross-referencing results from the previous two components. Rapid analysis of staff interviews and leader surveys were coded according to a spectrum of perceived effort and impact (28). Results identified seven priority strategies, primarily enacted by the CEDI support system (37) (SW, CC) to target behavioral change in the delivery system (medical assistants and clinicians). See Table 4 for a breakdown of the proposed strategies [described with best-practice language from a common taxonomy of implementation strategies (26)], with corresponding change objectives, and specification per best practice guidelines for describing implementation strategies (25).

**Table 4:** Implementation strategies generated by implementation mapping.

**Strategy**	**Change objective**	**Specification**
Change EHR record systems Use data experts	Skills for adopters and implementers	Data experts at academic partner and FQHC will add optional 5A's-concordant smart forms and patient after-visit summaries on a trial basis (3 months) to the EHR that will be activated by tobacco fields already being used.
Remind clinicians	Skills for implementers	Automatic reminders will be added on a trial basis (3 months) to the EHR to address tobacco use. These will not be mandatory to complete.
Develop academic partnerships	Attitude and knowledge of adopters and implementers	The IM protocols and materials will be co-produced by the FQHC and academic partner at the beginning of the implementation period.
Work with educational institutions Develop educational materials Conduct educational meetings	Attitude, knowledge, and skills for implementers	The academic partner will create 5A's educational materials and facilitate educational sessions with FQHC clinicians and staff over the course of 3 months during catered lunch breaks.
Auditing and feedback	Skills for adopters and implementers	Data experts at the academic partner and FQHC will create an audit tool for supervisors to easily pull tobacco measures, prescriptions, and quitline referrals by clinic.

### Step 4: Produce implementation protocols and materials

This step aims to enact the implementation strategies through content development. For the strategy of incorporating elements of the 5A's intervention into the EHR, the implementation support team enlisted assistance from EHR analysts (from the FQHC and academic affiliate) and tobacco cessation experts from the academic affiliate. This team created pharmacy order sets within the EHR to speed clinician access to different prescription options for the Arrange step while completing the patient visit, and a sample after visit summary page—including cessation tips and guidance on how to use medication therapies—to be provided to patients. The team also created a data analytic strategy for pulling summaries of tobacco users and completion of 5A's steps (i.e., advice to quit, pharmacotherapy prescriptions, printing patient after-visit summaries, and referrals for behavioral treatment).

Educational materials for adopters included workflow diagrams and detailed flowcharts of decision points and documentation requirements for each of the 5A's steps. Different flowcharts were created for the adult and pediatric clinics to account for differing algorithms. These specified when in the patient visit the innovation was to be enacted (i.e., after taking vital signs) and suggested prompts to start the conversation with patients (e.g., during the Advise step a clinician could state “Can I share with you why I think it is important to your health for you to stop using tobacco products, and how I can help you?”). To enhance usability, these flowcharts were limited to one page with clear font and large text.

Faculty from the local academic affiliate (which runs a tobacco treatment specialist training program) provided sample lecture slides and quick-reference handouts for the development of clinician and staff educational materials.

### Step 5: Evaluate implementation outcomes

Since this study was an implementation pilot, outcomes focus on the broad feasibility of the IM process (11). The IM process took approximately 12 months. True to the iterative nature of IM, feedback from FQHC staff and leadership informed revisions of the implementation package. The process of conducting the needs assessment and defining determinants, objectives, and strategies was feasible with a small, collaborative team.

Per anticipated implementation outcomes, in the early IM stages staff interviews indicated a majority were either in favor (67%) or neutral toward (17%) implementing the computerized 5A's process, indicative of good acceptability. Similarly, the majority (86%) of clinic leaders were in favor of proceeding with the plan to implement the computerized 5A's, indicative of adoptability. However, during review of the final implementation package, FQHC executive leadership expressed reductions in willingness to integrate the 5A's intervention package as shown in the protocols and materials.

In meetings following the development of the implementation package, leadership and clinicians involved in the CEDI team reported that the COVID-19 pandemic had caused significant strain on the FQHC, as well as its staff and clinicians. Specific barriers to proceeding with implementation were consistent with those originally voiced by both staff and clinic leadership during IM steps 1 and 2. Although in the early steps of IM limited clinical appointment time and risk of staff burnout were perceived as manageable barriers to implementation, they later became salient to organizational leadership as barriers—and perhaps insurmountable due to the pandemic.

Given the limited time frame of the study funding period and competing priorities of FQHC staff and leadership, further work on revising the implementation package has not been possible. Changes to the EHR have not yet been made, trainings have not been completed, and requests have not been made to the medical staff to change care or documentation of tobacco cessation. Despite promising early indicators of acceptability and adoptability, at time of publication the actual implementation outcomes for implementers (medical assistants and clinicians) were unfortunately not achieved.

## Discussion

The five steps of implementation mapping were conducted with an FQHC for implementing a computerized tobacco cessation intervention. Despite following IM recommendations and achieving early implementation outcomes of acceptability and adoptability, the intervention was not adopted. While not yet successful in its intended efforts, this project offers important lessons for future use and improvement of IM application in community clinics.

### Lessons learned

The most significant barrier to achieving intended outcomes is not accounted for by standard implementation methods: a global pandemic. CFIR and other implementation models recognize the vast effect of broad external factors on implementation success (24, 37). Changes in outer context (local, national, and global) affect the inner context (individual, team, organizational). Without data to investigate the salient factors after the onset of the COVID-19 pandemic, the authors surmise that the substantial effect of the pandemic on healthcare organizations altered FQHC staff perceptions about 5A's priority and compatibility. This is likely due to rapid rollouts of new disease mitigation processes and a sudden increase in telehealth technology needs. Additionally, the determinants may have been affected by the changing financial, temporal, and logistical resources of the FQHC. The initial needs assessment was instrumental for understanding the performance objectives and change objectives and developing the initial implementation package, but a repeat assessment of determinants could have assisted in understanding evolving barriers to uptake and optimal strategies to address them.

Identifying implementation strategies requires assessing and addressing both individual and organizational-level components, a point reinforced by successful IM examples (8). While this project developed multi-tiered strategies by involving multiple stakeholders and conducting IM as an iterative process, logistical barriers preventing this project from including the intended recipients of this innovation. Patients were unavailable for participation in the project during the early phases of the COVID crisis. This omission highlights that patient perspective may be a critical component for IM success.

Additionally, this IM process took 12 months. Compared to other examples that unfolded over several years (8) the time elapsed may have been too brief to achieve practice utilization. In interviews, FQHC staff noted the need for time and resources to adopt and scale this innovation. This highlights the stressors of using limited external funding, which follows grant cycles and stipulations, and may require much greater funds to follow the full implementation process through to the maintenance phase. Here, the external support was limited to one year. It is well documented that successful implementation requires long-term support and strategies (38), which requires funders' long-term investment of implementation projects (39, 40). While external funding sources provide critical supports for knowledge transfer, there remains a lag between the significant resources needed for successful implementation (38) and the structure of funding mechanisms (39, 41).

#### Implementation mapping

IM remains a promising and feasible method for effectively planning and strategizing implementation efforts (8–10). The method is continuing to be tested and improved. Several large studies using IM are planned or underway (4, 6, 7), which will further describe and refine the process. While the evidence base grows, the practice-based evidence supplied here bridges implementation practice to implementation science.

Based on this project's findings, IM does not sufficiently guide how to manage contextual changes that occur over time. It is well documented that determinants display variable salience across implementation stages (42–44). In our example, during early IM steps the FQHC adopters and implementers reported enthusiasm for the innovation. Yet the relative priority may have changed, likely due to the COVID-19 pandemic shifting organizational needs. Accounting for external disruptions to the implementation process is necessary for both building organizational resilience and enhancing implementation success (45). Relatedly, regardless of changing priorities, preliminary work identified that some strategies are relevant in earlier versus later stages of change (46). Although IM is proposed as an iterative process, it is unclear when and how often users should re-assess determinants and revise strategies. This is likely to vary by context, however—as originally suggested by the IM developers (3)—implementation practitioners would benefit from expanding the literature on how IM can be synchronized with frameworks that account for other influences on the implementation process. Frameworks of implementation stages (47–49) may be critical supplements for IM. Timely re-assessment of determinants and strategy selection—with appropriate resources for doing so—could have assisted in effectively adapting implementation protocols for the rapidly changing FQHC context.

Similarly, given resource constraints in certain care settings, *prioritization* of change objectives is an essential element that should be added to the IM process. Translating determinants into change objectives is a critical step. This effectively decides, across roles and systems, which key elements are needed to affect change. Here, relative priority and compatibility of the 5A's were identified as highly important in the data. However, among the actions prescribed by Table 3, which are most influential? Ideally, implementation protocols would enact strategies to address all the change objectives, yet this is not feasible in practice. IM developers suggested one determinant framework of organizational readiness may aid in the second and third steps (3). Since the initial IM publication, guidelines (5) and tools (45) for systematically prioritizing determinants have been developed for this readiness framework along with proposals to validate readiness measures in FQHCs (50). Relatedly, models accounting for the behaviors, capabilities, opportunities, and motivations (52) of staff could sharpen assessment of determinants and match them appropriately to change objectives. Use of these instruments by implementation practitioners are consistent with IM recommendations to be both integrative and iterative.

## Conclusion

Although this pilot did not result in adoption of the computerized 5A's intervention, IM was feasible to conduct in an FQHC with limited resources. Future IM use should allocate more than one year for reaching intended outcomes and re-assess determinants and change objectives at regular intervals. IM users would benefit from explicit instructions for when to re-assess determinants and how to merge IM with other implementation frameworks. These considerations may improve ability to reach sustainment in future projects.

## Data availability statement

The raw data supporting the conclusions of this article will be made available by the authors, without undue reservation.

## Ethics statement

The study protocol was reviewed and approved by Duke University Health System Institutional Review Board. The need to obtain written informed consent was waived by the review board. Prior to beginning study procedures, the participants provided either verbal consent or reviewed a consent script in digital format.

## Author contributions

This work was conceived, designed, and executed by SW and CC with assistance from HE, KP, JD, and PC. AD analyzed data and drafted the majority of the manuscript. HE, KP, JD, CC, and SW provided substantial revisions. Additionally, PC provided design oversight, consultation, and critical comments. All authors contributed to final read and provided approval for publication of the content.

## Funding

This work was supported by resources provided by the National Center for Advancing Translational Sciences (UL1TR002553) and by a Duke School of Medicine Department of Psychiatry & Behavioral Sciences Health Inequities Research Pilot Grant.

## Conflict of interest

The authors declare that the research was conducted in the absence of any commercial or financial relationships that could be construed as a potential conflict of interest.

## Publisher's note

All claims expressed in this article are solely those of the authors and do not necessarily represent those of their affiliated organizations, or those of the publisher, the editors and the reviewers. Any product that may be evaluated in this article, or claim that may be made by its manufacturer, is not guaranteed or endorsed by the publisher.
